# Awareness of and Attitudes Toward User Involvement in Research on Aging and Health: Protocol for a Quantitative Large-Scale Panel Study

**DOI:** 10.2196/17759

**Published:** 2020-09-21

**Authors:** Maya Kylén, Steven M Schmidt, Oskar Jonsson, Björn Slaug, Susanne Iwarsson

**Affiliations:** 1 Department of Health Sciences Lund University Lund Sweden

**Keywords:** partnerships, public involvement, older people, UserAge program, user participation

## Abstract

**Background:**

User involvement is a requirement of most research funders. There is a growing body of literature exploring the benefits and challenges of user involvement in research, but such studies are scarce in the field of aging and health. Moreover, the majority of such research is qualitative, which limits the generalizability of results. The UserAge panel study will be instrumental in expanding knowledge that will benefit the quality and impact of user involvement in future research.

**Objective:**

The aim of this study is to determine the awareness and understanding of and attitudes toward user involvement in research among different categories of knowledge users and researchers over time.

**Methods:**

A panel study will be implemented with 3 different categories of knowledge users (people aged 60 years and older, informal carers, and professionals in health care and architecture) and researchers in aging and health. A professional survey company will collect data from all samples in parallel. Potential participants will be asked to complete the survey via telephone or online, or participants can request a paper survey to be sent to them in the post. A draft set of questions on attitudes and behavioral patterns related to research utilization and user involvement in research was compiled based on existing literature and input from the research team. Using a participatory approach, we engaged a user forum, where 8 older people and 3 researchers jointly refined the survey for time/length to complete, terminology, readability, and context. Data collected via the internet or telephone will be automatically processed, and data collected on paper forms will be entered in machine-readable forms. The survey company will store all data and deliver the quality-controlled database to the university for further storage. Analyses of frequencies and measures of central tendency will be used for descriptive purposes. To compare groups, state-of-the art statistical analyses will be used.

**Results:**

Data collection for the first study wave started in September 2019 and will be completed in spring 2020. Data will be ready for analysis following cleaning and quality control, which started during summer 2020 and will be completed autumn 2020. We anticipate the data collection for the second study wave to start in September 2021.

**Conclusions:**

This is the first quantitative large-scale panel study focusing on trends in attitudes toward, awareness of, and knowledge about user involvement in research on aging and health in Sweden. The results will generate new and important knowledge to advance the understanding of user needs and preferences as well as the relevance of user involvement in research on aging and health.

**International Registered Report Identifier (IRRID):**

DERR1-10.2196/17759

## Introduction

### Background

The rising proportion of older people in the population has increased the demand for new solutions and targeted public and welfare services. Among these are aging-related policies addressing infrastructure, health care, social services, and housing [1]. In order to develop efficient strategies and ensure that research on aging and health is effectively translated and promptly put into practice, policy makers underscore the need to involve different categories of knowledge users in the research process [2,3]. Although patient and public involvement is increasing in fields such as mental health and disability [4-6], aging and health research is lagging behind. There is limited knowledge about the awareness of and attitudes toward user involvement among aging and health researchers and the different categories of knowledge users who could be involved. Such studies have typically involved representatives of nongovernmental, senior citizen, and patient organizations. Older people are a heterogeneous group with diverse preferences and needs. They want the opportunity to influence the products and services they need and would like to choose from a variety of options, but little is known on the opinions about user involvement in research on aging and health in the general population of older adults.

According to the World Health Organization (WHO) [7], “knowledge users” represent all those who are interested in or able to directly or indirectly benefit from aging and health research results. For example, knowledge users may include older people in the general population or those with specific needs; informal carers; patients; health care, social services, and industry professionals; and public agency, policy makers, and interest organization representatives. Knowledge users are those in a position to identify problems and act on research recommendations [8]. User involvement in this context is seen as *active* involvement of knowledge users as partners in any stage of the research process and not as research subjects or study participants in the traditional sense [9]. Hence, user involvement in this study protocol paper refers to research that is conducted “*with* or *by*” knowledge users rather than “*to*, *about,* or *for*” them [9]. User involvement may help researchers to understand some of the complex and context-specific nature of health and social problems experienced by older people [10] as well as practice and policy change [9], and contribute to the development of age-friendly services and communities [11]. Yet, such studies are few, and we know very little about how different categories of knowledge users and researchers perceive user involvement as a phenomenon in this field of research.

Systematic literature reviews have reported many ways in which user involvement impacts research processes and outcomes as well as the people involved [12-14], but the evidence has been criticized as being weak and unreliable [15]. A main reason for this criticism is that research on user involvement, so far, has been dominated by small-scale qualitative studies [16,17]. This limits the generalizability of results, and the accumulation and building of knowledge for the future is slow. Although qualitative studies are highly commendable to gain in-depth knowledge, quantitative studies are necessary to establish a broad knowledge base that can be used to make inferences about relevant populations.

Research on attitudes toward user involvement has primarily focused on patients and clinicians [18] rather than the general population, including societal sectors other than health care and social services and researchers. Because the ongoing demographic shifts extend the demands on the delivery of welfare services, broader and additional categories of knowledge users such as representatives from the complex field of housing construction and provision need to be involved in research partnerships [19,20].

Qualitative research with a small number of participants reveals that health researchers recognize the potential benefits of user involvement, but the procedures are challenging [21]; researchers as well as knowledge users have highlighted the need for more training in this area [22]. Moreover, concerns have been raised about the strong political imperative to involve knowledge users, which may cause problems if researchers do so out of necessity rather than thoughtful commitment [23]. Thus, systematic approaches based on state-of-the-art methodology are needed to address these issues in a manner that generates reliable and valid results.

Involving knowledge users actively in the research process is a complex and context-dependent exercise [24]. A more thorough exploration of attitudes toward user involvement from both knowledge users’ and aging and health researchers’ points of view can help to create more favorable conditions for such studies to succeed. Furthermore, solid and reliable evidence is needed to determine which categories of knowledge users should and could be involved in research on aging and health. Expanding such knowledge will promote research partnerships as well as inform policy makers and funding agencies about how to increase the quality and impact of user involvement in research on aging and health. The UserAge panel study will be instrumental in this vital knowledge quest.

### UserAge and the Panel Study

UserAge is a 6-year research program designed to expand the understanding of user involvement in research on aging and health [25], with the integration of researchers and different categories of knowledge users at the core. The program is implemented as a national 4-university endeavor with international collaboration. Currently, 19 researchers and 4 PhD students representing different disciplines and research fields (eg, cognitive science, design sciences, gerontology, health sciences, philosophy, psychology, and sociology) are engaged in the program. UserAge is based on previous and ongoing research with various forms of participation involving different categories of knowledge users (eg, people in the general aging population; older people with frailty; older migrants; older people with substance abuse problems; informal carers; interest organization representatives; health care, social services, construction sector, transportation sector, and tech sector professionals; research institutes; public agency officials; and policy makers). The aim of the UserAge program is to enhance the execution of high-quality research and to increase the knowledge about the added value stemming from user involvement in the research process. The panel study described in this protocol paper is one of the empirical projects of the program.

The panel study has 2 primary aims addressed toward different categories of knowledge users and researchers in aging and health in Sweden:

1. What are the awareness and understanding of and attitudes toward user involvement in research?
2. Are the awareness and understanding of and attitudes toward user involvement in research changing over time?

## Methods

In collaboration with the program’s User Board, we established a user forum with 8 members representing older people, 2 researchers (authors MK and OJ), and 1 doctoral student. The user forum provided input into the final methods used for recruitment, procedures for data collection as well as content for the questionnaire.

### Study Design

The study design is a panel study with a baseline survey and at least 1 follow-up survey. With a longitudinal design such as a panel study [26], trends in attitudes toward and awareness and knowledge about user involvement in research on aging and health will be elicited over time. Panel studies also allow for replacement of participants when lost at follow-up. Collecting data at different points in time has the advantage of revealing shifting attitudes and patterns of behavior [27] that might go unnoticed with other research designs.

The baseline data collection period is autumn 2019 to spring 2020. The first follow-up will take place in 2021-2022; pending additional funding, a second follow-up is planned 2 years thereafter, that is, 2023-2024. Based on the results of the baseline survey and findings from other projects within the UserAge program [25], the survey questionnaires and data collection procedures will be refined. While all core questions will be used repeatedly, a few items might be added or slightly modified.

### Participants and Recruitment Strategy

The targeted study sample sizes for the different categories of knowledge users and researchers (in total, N=1500, see Table 1) are as follows:

1. People aged 60 years and older (60+ sample, n=1200)
2. Informal caregivers (carers sample, n=100)
3. Professionals within health care and architecture (professionals sample, n=100)
4. Researchers in aging and health (researcher sample, n=100)

**Table 1:** Overview of study samples, inclusion criteria, recruitment, and data collection methods.

Methods	Panel study samples (N=1500)			
	60+	Carers	Professionals	Researcher
Sample size, n	1200	100	100	100
Participants	People from the general population	Informal carers	Professionals	Researchers within aging and health
Inclusion criteria	Aged 60 years or older	Aged 60 years or older, or caring for someone who is 60 years or older	Professionals within health care or architecture, with relevance for aging and health	Experience of research on aging and health
Recruitment strategy	Random sample selected from national population registry data, stratified by sex	Convenience sample recruited from the organization Carers Sweden	Convenience sample recruited from a memory clinic (health care professionals) and advertisement in a professional newsletter (architects)	Convenience sample recruited from partner universities affiliated to the Swedish National Graduate School for Competitive Science on Ageing and Health and the Swedish Gerontological Association
Data collection method	Web-based survey Telephone survey Postal survey	Web-based survey Telephone survey Postal survey	Web-based survey	Web-based survey

The age range for inclusion (60 years and older) was set in consensus discussions among members of the user forum. Representatives of older people proposed this relatively low age with arguments that it takes time to contribute to societal change in many aging- and health-related issues, such as preventative work and housing for later life. The researchers saw potential benefits to include not only today’s but also tomorrow’s senior citizens. With the 60+ group, we are striving for a representative sample, with participants randomly selected from the Swedish national population registry, stratified by sex. Based on population data from Statistics Sweden (2017), there are approximately 2.57 million (52.8% women) people aged 60 years and older. Using a confidence level of 95% and a margin of error of 4, we estimate a total sample size of 1200 (600 women and 600 men) to be nationally representative [28]. Based on recent experiences with surveys in Sweden targeting older people [29], we expect a 50%-60% response rate. Thus, we will draw an initial random sample of N=2400. Using established techniques to substitute for dropouts over time [30], the goal is to maintain the sample size for the 60+ sample longitudinally. Additionally, during recruitment of wave 2, to achieve a representative sample, we will oversample those groups of the population who were under represented during wave 1. Recruitment will continue until the targeted number of participants within each stratum complete the survey.

The carers sample (n=100) will be a convenience sample of informal carers recruited from Carers Sweden’s (a non-governmental organization that supports carers, independent of any political or religious affinity) member list. In this study, informal care refers to unpaid care provided by significant others such as family, close relatives, or neighbors. Anticipating a 33 % response rate based on previous experiences, carers Sweden will post invitations to 400 of their members. Only carers aged 60 years or above or caring for someone who is 60 years or older will be included. Recruitment will continue until 100 informal carers have responded.

The professionals sample (n=100) will be a convenience sample. One sample will be health care professionals from a university hospital memory clinic, which has primarily older patients with various symptoms of cognitive decline such as dementia. The operations manager for the memory clinic will send out an invitation to the survey to employees, which includes nurses, occupational therapists, physiotherapists, and medical doctors. The other sample will be architects, interior architects, landscape architects, and spatial planners recruited from a professional organization with 13,000 members. We will advertise the survey once in their weekly newsletter. Recruitment for the professionals sample will continue until 100 professionals have responded.

The Researcher sample (n=100) will be recruited through the national partner networks of the Swedish National Graduate School for Competitive Science on Ageing and Health (SWEAH) and the Swedish Gerontological Society (SGS). An invitation will be emailed to members/affiliates of both organizations. Recruitment will continue until 100 researchers have responded.

### Procedure

A professional survey company (Kantar Sifo) with longstanding expertise will implement the data collection. Each sample will receive an invitation letter that describes the project and information about the participants’ role and rights if they choose to participate. The invitation letter and survey for study samples 1-3 are in Swedish, while the survey for the Researcher Sample is in English. Samples 1-3 will receive information about the opportunity to sign up for interest to get involved in other parts of the UserAge Program (eg, engage in user fora to discuss and test research ideas, methodologies, evolving results, and practical solutions with researchers; participate in qualitative studies on user involvement in research). Instructions describe how to complete the survey via telephone, at an online secure web-page, or through a postal survey, as well as how to decline participation. The professionals and the researchers sample participants will only be able to respond to the survey online. Approximately 2 weeks after the letters are posted, trained interviewers from the survey company will call potential participants who have not completed the survey online or have not declined participation. Once telephoned, potential participants can choose one of the following options:

1. Request to be called at a different time/date to complete the survey
2. Request to complete the survey online
3. Request a paper version of the survey be sent to them in the post
4. Complete the survey immediately via telephone
5. Decline to participate

Data collection for all samples will be conducted in parallel. For potential participants who decline to participate, the survey company will ask them an open-ended question about why they have declined. This information will be used to identify risks to the representativeness of the 60+ sample.

### Panel Study Questionnaires

As a first step in an iterative process, the research team developed a draft set of questions on attitudes and behavioral patterns related to research utilization and user involvement in research on aging and health. Questions were based on existing relevant instruments [31-33] and iterative input from core researchers in UserAge. The user forum was engaged to jointly refine the survey for content, time/length to complete, readability, tone-of-voice, and understandability and to put questions into context. Members of the user forum provided input on the development of the invitation letter and method of survey implementation (phone, paper, and web). The user forum involved three 3-hour sessions during a 2-month period. Furthermore, a web seminar with UserAge researchers provided additional input, resulting in a core set of questions for the 60+ sample (see Table 2 for examples of questions included in the survey). The survey for the carers sample and professionals sample is based on the same core questions but modified to fit the perspective of the respective category of knowledge users. The survey for the Researcher Sample was compiled based on existing literature and feedback from UserAge researchers, as well as a set of comparable core questions. All 4 surveys included demographic questions such as age, sex, and level of education. In addition, the surveys for samples 1-2 included questions on perceived health and functioning. Prior to the data collection, a pilot study will be completed for the 60+ sample (n=25), carers sample (n=15), and researcher sample (n=15). Following this, the number of invitations to reach the targeted study sample sizes and questions may be slightly modified. As we intend to limit the time burden on participants to a maximum of a 10- to 15-minute phone survey, items may be removed from the survey based on the pilot study experiences and findings.

**Table 2:** Key sections and exemplar questions and responses translated to English.

Section and examples of questions in the survey		Response alternatives and scales
**Awareness and understanding**		
	Do you know that you can participate actively in the actual conduct of research? For example, give comments on questionnaires, membership in user boards, help to recruit study participants, or disseminate research results.	Yes/No/Don’t know
	How interested are you in research on aging and health?	5 response alternatives ranging from “not at all” to “very much”
	Would you consider actively participating in research on aging and health?	Yes/No/Maybe
	If you had the opportunity, how likely is it that you would want to participate by... Contributing to the planning and design of research projects. Being part of a user board, reference group, user panel or similar. Carrying out tasks in research projects. Analyzing the data produced. Disseminating research results. Contributing to an application for research funding.	5 response alternatives ranging from “not at all” to “very much”
**Attitudes**		
	People who are affected by research have a right to have input on what and how research is undertaken.	4 response alternatives ranging from “strongly disagree” to “strongly agree”
	Due to the fact that users^a^ in general have valuable life experiences, they should be actively involved in research on aging and health.	4 response alternatives ranging from “strongly disagree” to “strongly agree”
	User involvement is a symbolic political initiative that has questionable value for the results.	4 response alternatives ranging from “strongly disagree” to “strongly agree”
	Users^a^ should be actively involved in any publicly funded research on aging and health.	4 response alternatives ranging from “strongly disagree” to “strongly agree”
**Facilitators and barriers**		
	Through which channels do you prefer to be informed about opportunities to participate actively in research on aging and health?	Checkbox and free-text options. Letter in post, advertisement or article in newspaper, internet/social media, personal phone call, email, SMS, TV/radio, public meeting/conference or lecture, advertisement on message board, or other (specify).
	What could motivate you to participate actively in research on aging and health?	Checkbox and free-text option. Getting priority to services (eg, health care, social care, services, housing); to feel important; to find out more about my situation; to contribute to society; you have nothing to lose; research should go forward; to connect with others in the same situation; being helpful to the researcher; getting better services; finding out what the study will lead to; the research is about something that you think is important; others (specify); or nothing, you do not want to be actively involved in research.

^a^Instead of the term “user,” Swedish terms defining the targeted category were used in samples 1-3: “private individuals” in the 60+ sample; “carers” in the carers sample; and “professionals” in the professionals sample.

### Quality Control and Data Handling

At the start of the data collection, a researcher (MK or OJ) will monitor 25 of the first 100 telephone surveys to ensure procedures are followed for data quality. Throughout the data collection, periodic spot checks of the telephone surveys will be conducted to ensure that the telephone interviews are administrated in accordance with the agreed procedures. In total, 5 % of all telephone interviews will be listened to, simultaneously in real time, by 1 of the researchers (OJ). Furthermore, after 10% of the surveys are completed, a data quality check will be conducted by the survey company to identify any systematic errors in the data collection or data entry process. Data collected via the internet or telephone will be automatically processed, and paper surveys will be entered in machine-readable forms. All data will be stored by the survey company in a secure database in compliance with The General Data Protection Regulations (GDPR). The survey company will conduct a manual quality control check on all paper surveys.

Upon completion of the data collection and quality control, the survey company will develop a set of weights (numerical coefficients) for the cases to adjust for any underrepresented segments of the population in the 60+ sample to ensure representativeness. The following key characteristics will be considered: sex (male/female), age (60/65-65/70-70/75-80/85+ years), and geographic regions. People with underrepresented characteristics will be given higher weights, if necessary, while overrepresented characteristics will be restrained.

The complete data file will be encrypted by the survey company and transferred to the researchers and stored on Lund University’s platform for storing, handling, and analyzing data in a high-secure way (LUSEC). Only project researchers will be able to access the data within LUSEC. All analyses will be conducted using statistical software available within this platform (eg, SPSS and STATA).

### Data Analysis

Frequencies and measures of central tendency will be used for descriptive purposes. Exploratory comparisons between the groups will be used to identify differences in awareness of, understanding of, and attitudes toward user involvement in research. To compare groups, appropriate analyses will be selected based on the type of data (ie, ordinal, nominal, and continuous). Although no power calculation was conducted for comparisons across samples, as most of the survey items are categorical (yes/no) or ordinal (4 or 5 levels), we estimated that the selected sample sizes would provide adequate cell sizes for analyses. Specific data analyses will be reported in the methods sections of future publications. When applicable, we will use statistical methods such as multivariate regression and methods to deal with repeated measurements. In all analyses, two-sided *P* values will be used at a significance level of .05.

### Ethics

Ethical approval has been obtained from the Ethical Board in Lund (No. 2018/986; Dec 2018). Participation in the study should not present any significant risks, as the questions are not expected to elicit any sensitive or emotional reactions from participants. Participation is voluntary and participants can withdraw from the study at any time.

The survey company staff are specialized in conducting telephone surveys and will undergo project-specific training. We will ensure that they repeat the purpose of the study prior to each survey, speak in a clear and polite manner, and give potential participants an additional opportunity to decline to participate. The participant's right to discontinue the survey at any time will be clearly stated. The survey company staff will explain that all data will be handled in accordance with GDPR standards, protecting unauthorized access. It will be clearly explained on the paper version, by the telephone interviewer, and on the web-based survey that completion of the survey constitutes informed consent to participate in the study.

### Availability of Data and Material

The datasets generated and analyzed during this study are not publicly available due to a data use agreement between Lund University and Kantar Sifo but are available from the corresponding author on reasonable request.

## Results

Funding for the larger UserAge program started in January 2017 and will continue for 6 years. The data collection for the first study wave started in September 2019 and will be completed in spring 2020. Data will be ready for analysis following cleaning and quality control, which started during summer 2020 and will be completed autumn 2020. As of submission of this protocol, we have enrolled the following samples:

1. n=881 in the 60+ sample. Based on lessons learned from the pilot study, the random sample was increased to N=3000
2. n=150 from the carers sample after additional referral sampling
3. n=65 from the researcher sample
4. n=11 from the professionals sample

We anticipate the data collection for the second study wave to start in September 2021.

## Discussion

This paper provides a detailed description of a panel study, which is a part of the 6-year UserAge Program [25]. The panel study addresses key questions about the awareness and understanding of and attitudes toward user involvement in research among different categories of knowledge users and researchers. Given that research about and with user involvement is dominated by qualitative approaches [24], our study makes a valuable contribution by generating quantitative data that can be used to make inferences about relevant populations. In order to create favorable conditions for future research, it is crucial for aging and health researchers to understand the awareness and understanding of and attitudes toward user involvement among older people and informal carers. Importantly, by including 3 different categories of knowledge users as well as aging and health researchers, the panel study will generate new knowledge about how different categories of knowledge users perceive user involvement as a phenomenon in a research area of high societal relevance.

Involving different categories of knowledge users, each with specific needs and prerequisites, in research comes with specific challenges [34] hitherto insufficiently explored and addressed in a rigorous manner. In the panel study, the 60+ sample and the carers sample can choose to complete the survey via telephone, an online web page, or a postal survey. This approach will give more people the possibility to participate in the study [35], that is, if they do not have access to the internet or are unable to fill out a paper survey themselves, the telephone survey might be a favorable alternative. We will learn important lessons to optimize forthcoming data collection waves.

Methodological issues are of significant importance [14] because research funders today often ask for user involvement in their calls for research proposals. Accordingly, it is important to enhance the knowledge about the challenges of involving knowledge users in research on aging and health and about how to best handle them. Based on the overall goals of the UserAge program, of which the panel study is one of the numerous empirical studies, the intention was to include professional groups represented in different parts of the program. We thoroughly discussed different strategies to recruit participants representing health care and social services as well as housing and planning sector professionals. However, we experienced substantial challenges in this recruitment process and ended up with a less optimal sample in the first wave of data collection. A limitation worth mentioning in this context is that for the professionals sample, researcher sample, and carers sample, we used convenience sampling. In Sweden, there is no national registry or other means to identify the members of these 3 populations to randomly sample. Although we could have attempted to sample from the member lists of different professional or labor organizations in the health care, social services, and construction sectors, we did not have the resources necessary to do so in a valid manner. Thus, this part of the panel study mainly served as an exercise to gain experiences for future data collection waves, and the sample sizes attained in the first wave suggest a need to reconsider the sampling strategy. Another noteworthy challenge is the difficulties to clearly identify the target populations (professionals, researchers, and carers), which partly explains why we did not calculate statistical power; accordingly, these results cannot be extrapolated beyond the respondents. With lessons learned about recruitment and response rates, we will be in a stronger position to raise the level of ambition for forthcoming data collection waves. Nevertheless, our approach to include different categories of knowledge users, including participants without experiences of user involvement, in a large-scale panel study focusing on trends in attitudes and knowledge about user involvement in research on aging and health in Sweden has not previously been applied.

In conclusion, to the best of our knowledge, very few—if any—results from larger studies exploring attitudes and experiences of user involvement in research among the general population of older adults have been reported. As such, the UserAge panel study will provide results that can be used to inform research funders and policy makers about the prerequisites needed to efficiently conduct research with user involvement. This can lead to more relevant findings to improve well-being in later life; improve the ability of research partnerships to benefit from diverse knowledge users’ local, lived, or applied knowledge; and jointly address the challenges of the aging society in the best possible way. Findings from the panel study may create conditions to improve approaches to involve knowledge users (eg, channels for recruitment, meet interests and expectations, handle barriers) to increase the quality and impact of research as well as give knowledge users participating in research a meaningful experience. In addition, knowledge derived from the panel study will contribute to the development of reliable and valid methodologies to evaluate research with user involvement.

## Abbreviations

GDPR: General Data Protection Regulations
SGS: Swedish Gerontological Society
SWEAH: Swedish National Graduate School for Competitive Science on Ageing and Health
WHO: World Health Organization
